# REPRODUCTIVE TOXICOLOGY: Study Associates PFOS and PFOA with Impaired Fertility

**Published:** 2009-04

**Authors:** Carol Potera

Recent decades have seen a substantial decline in the number of children being born to women in developed countries. Much of this decline is likely attributable to sociocultural changes and increased access to birth control. But some studies suggest that exposure to environmental pollutants may play a role, perhaps by impairing fecundity, the ability to bear live children. Now a study by researchers at the University of California, Los Angeles (UCLA) has found that women who took longer to become pregnant were more likely to have higher blood levels of two common perfluorinated chemicals, perfluorooctane sulfonate (PFOS) and perfluorooctanoate (PFOA).

PFOS and PFOA are persistent manmade pollutants widely found in fabrics, carpets, food packaging, shampoo, nonstick cookware, insecticides, fire‐fighting foam, and other household products, as well as in industrial surfactants and emulsifiers. Potential major sources of PFOA in the environment include the degradation of fluorinated alcohols used in these products. Animal studies have associated both PFOS and PFOA with increased pregnancy losses and disruption in sex hormone homeo‐stasis and sexual maturation. Although previous studies have linked pollutants such as pesticides and polychlorinated biphenyls with delayed time to pregnancy, the UCLA study is the first to demonstrate a similar relationship with perfluorinated chemicals.

In the current study, the researchers measured maternal blood concentrations of PFOA and PFOS in 1,240 pregnant women who participated in the Danish National Birth Cohort from 1996 to 2002. Blood samples were drawn from weeks 4 through 14 of pregnancy, and women were asked at approximately week 12 how long it had taken to conceive and whether the pregnancy was planned.

Median maternal blood levels of PFOA and PFOS were 5.3 ng/mL and 33.7 ng/mL, respectively. Women were assigned to four quartiles of exposure, with the lowest quartile serving as the reference group. Women in the three higher quartiles were about twice as likely to have taken longer than 12 months to achieve pregnancy or to have required infertility treatments to become pregnant, compared with the reference group. They were also slightly more likely to report irregular menstrual periods. The results, which are slated to appear in the May 2009 issue of *Human Reproduction*, were published online 28 January 2009.

The blood concentrations measured in the Danish women were similar to those found in other Western populations, “so the findings probably [are applicable to] other affluent Caucasian societies,” says study leader Jørn Olsen, chairman of the Department of Epidemiology at the UCLA School of Public Health. However, “it’s important for someone else to replicate the study in another group of women,” he notes. It’s unknown how PFOS and PFOA may contribute to infertility; it is possible the chemicals interfere with sex hormones, delay ovulation, or contribute to unrecognized miscarriage (the authors of the 2004 text *Clinical Gynecologic Endocrinology and Infertility, 7th Edition* estimate that 20–40% of women miscarry before they even realize they are pregnant).

The UCLA study “is the first study ever in humans on this topic, to the best of my knowledge,” says David Savitz, director of the Disease Prevention and Public Health Institute at Mount Sinai School of Medicine in New York City. The study “gives a limited first look at the issue,” Savitz says, “but does encourage continued evaluation of the reproductive toxicity of PFOA and PFOS.”

In 2006 the U.S. Environmental Protection Agency (EPA) asked manufacturers to voluntarily reduce PFOA emissions and product content by 95% no later than 2010 and to eliminate the chemical by 2015. Olsen says the new data could help strengthen arguments to eventually regulate PFOA and PFOS or phase them out. Meanwhile, a study by Antonia M. Calafat and colleagues at the Centers for Disease Control and Prevention, published in the November 2007 issue of *EHP*, found significant reductions in human blood levels of PFOA and PFOS (25% and 32%, respectively) from 2003 and 2004 compared with data for 1999 through 2000. The authors of that study suggested that successful efforts by government and industry to reduce usage and emissions of these chemicals are the most likely reason for the downward trends.

